# Diagnostic outcome of two different CT-guided fine needle biopsy procedures

**DOI:** 10.1186/1746-1596-2-31

**Published:** 2007-08-23

**Authors:** Lutz Welker, Reyhan Akkan, Olaf Holz, Holger Schultz, Helgo Magnussen

**Affiliations:** 1 Hospital Großhansdorf – Centre for Pneumology and Thoracic Surgery, Großhansdorf, Wöhrendamm 80, D-22927 Großhansdorf, Germany; 2 Research Center Borstel, Clinical and Experimental Pathology, D-23845 Borstel, Germany

## Abstract

**Background:**

CT-guided fine needle bioptic procedures (CTFNP) are characterised by low invasiveness, precise sample collection, a high diagnostic efficiency and support a rapid diagnostic process. A number of different fine needles and bioptic procedures are mainly used for tumour diagnostics today.

The aim of the present study was to characterise the most important technical issues of fine needle bioptic procedures. In addition, we directly compared the diagnostic outcome and reliability of the most commonly used Rotex Screw Needle – (RSN) and Yale Needle – (YN) bioptic procedure.

**Methods:**

In an experimental part of the study, using pig spleen, we measured the maximum number of sampled cells using different needles and aspiration volumes.

For the clinical questions we analysed all consecutive 340 patients in which CTFNP were performed between 1/97–12/05 in the hospital Grosshansdorf. We evaluated the number of adverse events based on all clinical available information and compared the cytological findings with the respective final diagnosis (confirmed: clinically n = 192, histologically n = 148).

**Results:**

Using the YN with at least some negative pressure we found a proportional increase of cell and tissue recovery with increasing number of needle movements.

A sensitivity of 78% and a specificity 98% indicate a high diagnostic outcome of CTFNP. We found no statistical significant difference in terms of sensitivity (80 vs. 68%) as well as complication rates (5.9 vs. 4.4%) between RSN or YN.

**Conclusion:**

As fine needle basically works like a cutting instrument, it is possible to raise the cell/tissue recovery. Keeping this in mind we found a high diagnostic outcome of CTFNP, which was largely independent of needle type and bioptic technique, and comparable with other conventional bioptic procedures.

## Background

Fine needle biopsy is a well established and a commonly used procedure in tumour diagnostics. In addition to the conventional endoscopic and percutaneous fine needle punctures there is an increasing interest in the use of flexible endosonographic ultrasound giuded fine needle aspiration biopsies. Based on the main technical principle the large number of different needles can be grouped into core-, cutting- and aspiration needles. Core- and cutting needle specimens are generally suitable for embedding and histological analysis, while aspiration needle specimen comprise most often smaller cell and tissue fragments, which should be evaluated by cytology. It has to be considered that most needles work by applying at least two functional principles; e.g. a YN both cuts and aspirates. Despite the long tradition of fine needle biopsy diagnostics, very little is known about the exact mechanisms that influence the quality and the outcome of the procedure.

The aim of the present study was therefore to answer the following questions: (1) Which are the technical factors relevant for successful use of RSN and YN? (2) What is the rate of adverse events using RSN and YN? (3) What is the diagnostic outcome of CT-guided RSN- and YN-punctures in benign and malign thoracic lesions?

## Methods

### Fine needles

**Figure 1:**
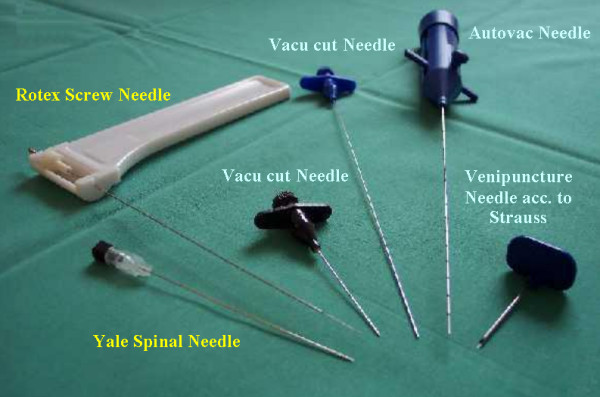
Different fine needle types.

The fine needles used in this study are shown in figure 1 and listed in table 1. The Rotex Screw Needle was supplied by Ursus Konsultant AB (Stockholm, Sweden), the Yale-Spinal-Needle by Becton Dickinson GmbH (Heidelberg, Germany), the Vacu-Cut-Needle and the Auto-Vac-Needle by BARD GmbH/Angiomed (Karlsruhe, Germany), and the Venipuncture Needle acc. to Strauss by B Braun (Melsungen Germany).

**Table 1:** Outer diameter, length and costs of different bioptic needles

Model	Ø in mm	Length in mm	costs in Germany
Rotex Screw Needle	0.8	90	58.90 €
Yale Spinal Needle	0.7	90	1.33 €
Vacu Cut Needle	0.8	100	18.41 €
Vacu Cut Needle	0.8	200	18.41 €
Autovac Needle	0.95	150	19.17 €
Venipuncture Needle acc. to Strauss	1.5	43	5.33 €

### Experimental part

First we measured the negative pressure obtained with different needles at different evacuation volumes (20 and 50 ml) and for different needle lengths. Next we determined the influence of needle handling (number of punctures) at maximum negativ pressure, on the sampled cell number using the YN and the RSN. These experiments were performed using fresh pig spleen tissue. The revealed cells were stained by trypan blue and total cell counts were assessed in a Neubauer chamber. For control cytospin slides were prepared from 50,000 cells (600 cpm, 15 min; Heraeus Sepatech Omnifuge 2.0 RS; Heraeus Sepatech, Hanau, Germany) and stained with May-Gruenwald-Giemsa for the cytological examination.

### Clinical part

The clinical part of the study was based on the data of all consecutive patients undergoing CT-guided fine needle bioptic procedures at the hospital Großhansdorf in the period from January 1997 to December 2005. In total we included 340 patients (243 m, 97 f). To determine the diagnostic outcome we compared the cytological result of 74 RSN- and 364 YN-biopsies with 192 clinically and 148 (132 specimens obtained during surgery) histologically verified final diagnosis. In addition, we determined the number of adverse events during and at least 24 hours after the procedure.

For each fine needle bioptic procedure the needle was inserted at least three times and a cytological analysis was performed for each of these samples. If at least in one of these samples malignant cells or cells of uncertain dignity were found the cytological diagnosis was malignant or uncertain. If in none of the samples malignant cells or cells of uncertain dignity were found the cytological diagnosis was benign.

### Data analysis

Median values and quartiles of cell numbers were computed and compared by Mann-Whitney-U test. Sensitivity, specificity and diagnostic reliability were computed as usual. Sensitivity and specificity were compared between different criteria by the Fishers exact test or the one-sided test of proportions, if appropriate. Statistical significance was assumed for p < 0.05.

## Results

### Experimental results

**Figure 2:**
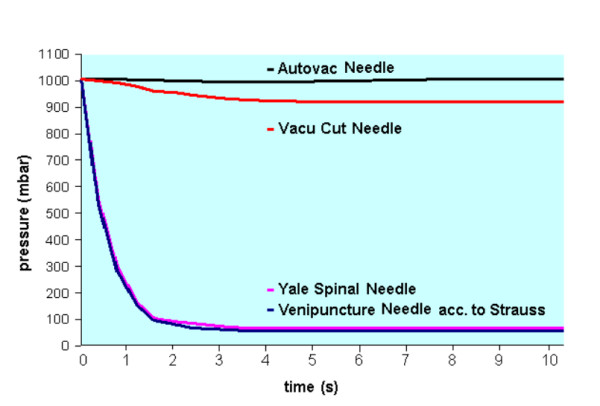
Pressure-time-curves with different needles and different needle length using evacuation volumes of 20 and 50 ml.

Although the majority of fine needles depend on the generation of a negative pressure there are remarkable differences between manufactors and needle models. Entering the Autovac and Vacu cut needles into pig spleen it was possible to generate only very limited negative pressure (always below 80 mbar, fig. 2). In contrast to these needles we measured a maximum negative pressure of 940 mbar and 931 mbar using the venipuncture needles acc. to Strauss or the YN respectively (fig. 2).

The main aim of fine needle biopsies is to collect a minimum required number of cells to allow a cytological analysis. With a single insertion of the RSN into a pig spleen we sampled a median (range) number 0.85 × 10^6 ^(0.83–0.87) cells. This needle needs to be pulled out after each bioptic procedure to release the cell specimen prior to the next insertion.

**Figure 3:**
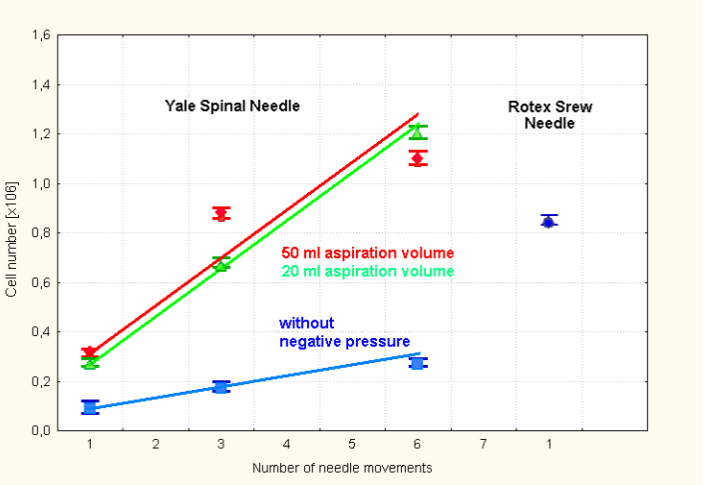
Pig spleen model – fine needle biopsy: number of harvested cells versus needle type and handling (median and range). Squares = YN without negative pressure, tri-angles = YN using 20 ml aspiration volume, diamonds = YN using 50 ml aspiration volume, circles = Rotex Screw Needle.

In contrast to RSN the YN does not need to be pulled out completely and can be insert into the tumour repeatedly by small needle movements. The number of these movements was directly proportional to the number of aspirated cells. Applying a sufficient negative pressure during these movements has further increase the efficacy of the YN. There were no differences between 20 ml or 50 ml aspiration volumes (fig. 3).

### Clinical results

We performed 438 fine needle biopsies in 340 patients, using the YN in 364 cases. In the majority of cases (n = 192) the final diagnosis was based on clinical findings and follow up. In 148 cases it was based on an independently performed histological analysis.

In 200 of 266 patients (75.2 %) with malignant tumours, the tumour was detected by cytology. In 61 of 62 patients with benign lesions the cytological analysis of fine needle bioptic material was able to determine the correct underlying dignity. Only in 12 patients neither clinical information (n = 10) nor histological analysis (n = 2) were able to find the correct dignity of the lesion.

Overall CTFNP of pulmonary lesions showed a sensitivity of 78 % and a specificity 98 % resulting in a diagnostic reliability of 77 % (table 2). CTFNP in the cases, in which the final diagnosis was confirmed clinically, showed a better sensitivity and specificity as compared to the histologically confirmed cases. However, neither in the clinical nor in the histologically validated cases a significant difference between YN and RSN could be detected (Fishers exact test p = 0.34; Tab. 2).

**Table 2:** CTFNP – Sensitivity, specificity and diagnostic reliability

Validated by:		n	sensitivity in %	specificity in %	diagnostic reliability in %
Clinical findings	192	84.6	100.0	83.9
YN	164	83.0	100.0	82.9
RSN	28	91.7	100.0	89.3
Histology	148	70.8	93.8	68.2
YN	114	68.0	92.3	66.7
RSN	34	80.0	100.0	73.5
Total	340	77.8	98.4	77.1

It is well known, that the success of a bioptic procedure critically depends on the sampling accuracy. Therefore the size of the lesion is important, and there should be a relationship between the volume of a tumour and the diagnostic reliability of the applied method.

In 132 resected tumours we were able to determine the exact tumour size and volume respectively. Below a tumour volume of 20 cm^3 ^we found a much lower sensitivity and lower diagnostic reliability as compared to larger tumours (Fishers exact Test p = 0.016; Tab. 3).

**Table 3:** Sensitivity, specificity and diagnostic reliability vs. tumour volume (resected tumours, n = 132)

Tumour volume in cm^3^	n	sensitivity in %	specificity in %	diagnostic reliability in %
<10	44	57.1	85.7	56.8
10–20	21	57.9	100.0	61.9
20–30	13	83.3	100.0	76.9
30–70	19	77.8	100.0	73.7
>70	35	84.8	100.0	80.0

### Rate of adverse events

The main adverse event of fine needle biopsy are a pneumothorax and intrapulmonal hemorrhage. In this study we observed a pneumothorax in 5.5 % of patients (9/163). All these were very small and only detectable by CT. Additional 16 patients suffered from larger pneumothorax which was detectable by conventional X-ray. Only in 7 of these patients it was necessary to apply a drainage. The rate of adverse events was not different between RSN- and YN-biopsies (5.9 vs. 4.4%) (Fishers exact test p = 0.291; Table 4).

**Table 4:** CTFNP – pneumothorax-rate RSN and YN (exclusively patients with histologically verified final diagnosis, n = 148)

	RSN	YN
Number of patients	34	114
Pneumothorax	rate % (n)	rate % (n)
According CT-findings	8.8 (3)	14.0 (16)
According Conventional X-ray	14.7 (5)	9.7 (11)
Thorax drainage necessary	5.9 (2)	4.4 (5)

## Discussion

In order to optimize the outcome of fine needle biopsy it is important to keep in mind, that all needles basically work like cutting instruments. Here we were able to show, that first of all the extend of needle movements raise the cell/tissue recovery and result in a high diagnostic outcome of CTFNP, which is even comparable with other conventional bioptic procedures. Both sensitivity and specificity, as well as complication rates were largely independent of needle type and bioptic technique.

In the recent years, cytology of the respiratory tract has been revolutionized by a high degree of sophistication in radiologic technology and sonography, making a precise visualization and localization of tumours in the lungs, bronchi, mediastinum or chest wall possible. Between 1997 and 2005 the files of the hospital Großhandorf cytology laboratory recorded a total of 9380 fine needle specimens (4847 rapid on site analysis). Basically any patient found to have a demonstrable radiolographic tumour is a potential candidate for a fine needle biopsy. A further decision on whether or not to proceed with this procedure is usually based on the morphologic evidence provided by prior cytologic and histologic specimens obtained from the respiratory tract and on the clinical relevance of morphological findings.

From all fine needle specimens several direct smears are prepared and air dryed. One of these smears will be chosen for rapid or standard Giemsa staining. Because of direct sampling within the tumour by a fine needle, these specimen most often contain large numbers of cancer cells and tissue fragments, leaving generally additional unstained slides for further routine- or immuncytological staining procedures. Despite that, the amount of material as well as the number of slides are usually limited and occasionally there are 9 patients (2.6 % in our case), in whom a cancer is suspected but can not be conclusively diagnosed by cytology alone.

In pneumology CTFNP are well established diagnostic procedures and in clinical practise a number of different needles are used. Despite of all technical differences all fine needles have in common that they function like a cutting instrument. In addition, turning and repeated puncture movements help to gain small cell clusters and tissue fragments. Although a applied negative pressure can assist in raising the amount of cells, this factor is of limited importance compared to the effect of cutting. This would explain that although a large differences in negative pressure between needle types exist (fig. 1), we found a similar diagnostic outcome with NN and YN. In addition, the number and handling of repeated punctures influenced the success of a fine needle biopsy, as we were able to show in our experiments with pig spleen (fig. 2).

The sensitivity and the diagnostic outcome of fine needle biopsy seems to be independent from the kind of morphological analysis (histology or cytology) and have been reported to range between 62%–83% (at a specificity of 100%) and 68%–85%, respectively [1-3]. Both with the YN and the RSN, which were used in this study, we found a very similar diagnostic outcome of 77 % as compared to the literature [4-6]. CTFNP therefore did not differ from conventional cut needle or forceps biopsies [7]. The diagnostic outcome, however, depends on the size of the target. In line with others [1] we also found that the increase of the diagnostic outcome corresponded to the tumour volume (table 3). This would also explain better values for sensitivity and specificity in group of patients with clinically verified tumours, as these tend to be larger in size as compared to those with histologically verified final diagnosis (table 2).

The most common complication of CTFNP are haemorrhage or the occurrence of a potentially drainage requiring pneumothorax. It is well known that the risk of such complications increase with the outer diameter of the needle. Using fine needles with a diameter > 1 mm leads to pneumothorax rates of more than 13 % [3,8]. Experienced clinicians, on the other hand, are able to keep the complication rate significantly lower, especially if needles with smaller calibre are used. In our study we recorded adverse events in only 6 % of all cases, with no differences between RSN and YN, which is in accordance with [4].

As shown in table 1 there are quite large differences in the costs of different needle types. We would like to emphasise that a clinician should always be working with a needle type he is familiar/experienced with, as, in the time of restricted hospital budgets, it could be argued that only low price needles should be used. With respect to the complication rate it is our experience that it increases with larger needles. In line with the literature we would therefore also recommend needles with outer diameter below 0.8 mm [8].

## Conclusion

In summary we would like to emphasize that a fine needle basically works like a cutting instrument, and repeated needle movements are of prime importance for both cells/tissue quantity and quality. In line with others we found that the diagnostic outcome of CTFNP was high. In addition we were able to demonstrate that the outcome was largely independent of needle type and bioptic technique, and comparable with other conventional bioptic procedures.

## Abbreviations

CTFNP – CT-guided fine needle bioptic procedures

RSN – Rotex Screw Needle

YN – Yale Needle

## Competing interests

The author(s) declare that they have no competing interests.

## Authors' contributions

LW and RA did the clinical fine needle punctures, cytological and data analysis. LW, OH and HM have been involved in drafting the manuscript and revising it critically. HS did the histological analysis. All authors have read and approved the final manuscript.
